# Supplementary material for: Optimization of Propagation of the Polish Strain of Aldrovanda vesiculosa in Tissue Culture
Source: Biology (Basel). 2022 Sep 23;11(10):1389. doi: 10.3390/biology11101389 (PMC9598617; doi:10.3390/biology11101389)
Supplement: Supplementary file 1 [file biology-11-01389-s001.zip › biology-1852518-supplementary.pdf]

Table S1. Content of photosynthetic pigments in *Aldrovanda vesiculosa* plant material from in vitro cultivation and from a natural site (Lake Łukie).

MS concentration	Nitrogen concentration	Chlorophyll *a* (μg·g^{-1})	Chlorophyll *b* (μg·g^{-1})	Carotenoids (μg·g^{-1})	Sum of pigments (μg·g^{-1})
MS	2N	-	-	-	-
	1N	-	-	-	-
	1/2N	394.14 def	156.94 cd	301.61 bc	853.33 de
1/2 MS	2N	132.01 f	71.76 d	443.60 a	646.67 e
	1N	373.44 ef	158.65 cd	178.52 d	710.00 e
	1/2N	531.25 cde	213.22 bcd	219.17 cd	963.33 cde
1/5 MS	2N	719.46 bc	280.66 abc	253.64 bcd	1250.00 bcd
	1N	950.41 ab	443.61 a	286.85 bcd	1676.67 ab
	1/2N	902.17 ab	335.34 abc	308.82 bc	1546.67 ab
1/10 MS	2N	913.74 ab	440.11 a	250.33 bcd	1606.67 ab
	1N	1067.20 a	410.09 ab	340.07 abc	1816.67 a
	1/2N	746.02 bc	312.02 abc	251.53 bcd	1306.67 a-d
Lake water		711.82 bcd	393.23 ab	355.19 ab	1460.24 abc

* Means followed by the same letter in columns do not differ significantly at $\alpha = 0.05$.

MS – Murashige and Skoog medium formulation, 1/2N, 1/5MS, 1/10MS – mineral salts diluted to 1/2, 1/5, 1/10; N – content of nitrogen compounds NH_4NO_3 and KNO_3 with concentration doubled (2N), unchanged (1N) or diluted twice (1/2N).
